# Thoracic and Renal Actinomycosis Requiring Complete Right Nephrectomy in a Costa Rican Female Child

**DOI:** 10.7759/cureus.6879

**Published:** 2020-02-04

**Authors:** Edward Segura-Perez, Rolando Ulloa-Gutierrez, María L Ávila-Aguero

**Affiliations:** 1 Pediatric Infectious Diseases, Hospital Nacional De Niños "Dr. Carlos Sáenz Herrera", San José, CRI

**Keywords:** thoracic actinomycosis, renal actinomycosis, nephrectomy, children

## Abstract

Actinomycosis is a relatively uncommon bacterial disease of childhood, especially when presenting as deep-seated infections. Only 10% of affected patients are younger than 18 years of age. In children, cervical actinomycosis is the most common form of clinical presentation, and among the abdominopelvic form, renal abscesses are rare. We describe an uncommon and severe case of a five-year-old Costa Rican girl with thoracic and renal actinomycosis who required a complete nephrectomy.

## Introduction

*Actinomyces spp*. are filamentous gram-positive bacilli that belong to the human commensal flora of the oropharynx, gastrointestinal, and urogenital tracts. Multiple different clinical features of actinomycosis have been described, and various anatomical sites can be affected. Actinomycosis frequently mimics malignancy, tuberculosis (TB), or nocardiosis and can spread continuously and progressively [1].

Actinomycosis mostly affects middle-aged adults. Pediatric actinomycosis is rare, accounting for less than 3% of all actinomycosis cases. In children, cervicofacial actinomycosis is the most common clinical presentation with 20% of pediatric cases occurring in the abdomen [2-3]. We describe a five-year-old female child with thoracic and renal actinomycosis who required both medical and surgical management.

## Case presentation

**Figure 1:**
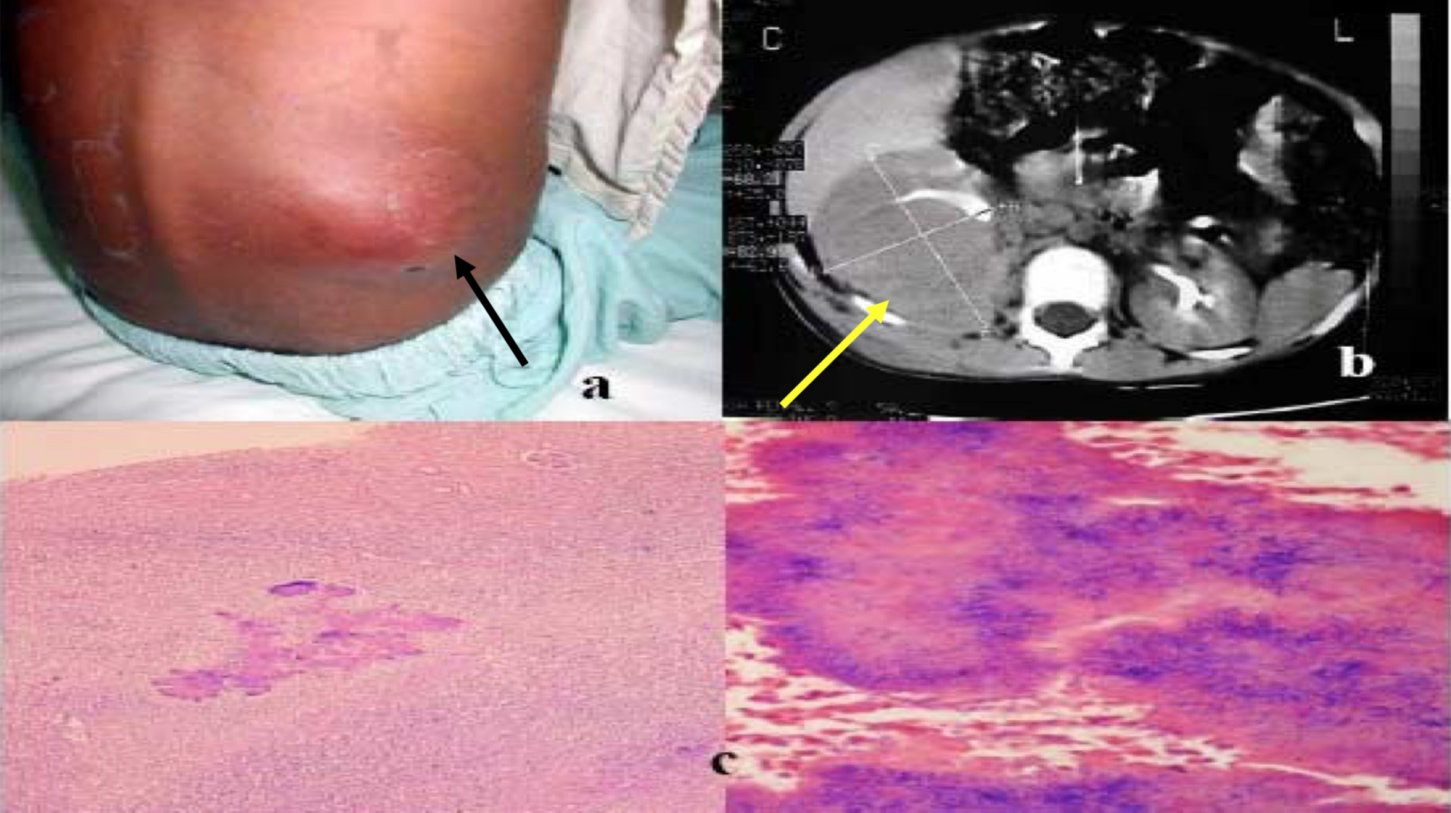
Injuries identified to physical examination and histology a) Lumbar mass without cutaneous fistula (black arrow); b) computed tomography scan with a heterogeneous mass involving the right kidney entirely (yellow arrow) with extension into the soft tissues; c) sulfur granules in renal sample

A five-year-old native Costa Rican female child was referred from a community hospital to our institution where she had been admitted one week prior complaining of a two-week history of a painful right lumbar mass, productive cough, and malaise. On admission, physical examination revealed an afebrile girl with severe periodontal disease, no heart murmurs, and decreased breath sounds in the right chest. No splenomegaly was found. However, the liver was palpable 3 cm under the right costal margin, and a 5 x 5 cm painful and fluctuating mass in the right lumbar area was documented (Figure 1a). Two lymphadenopathies were visible on the right hemithorax and axilla but there was no evidence of a cutaneous fistula. A complete blood count revealed microcytic anemia (hemoglobin 7.6 g/dL) and leukocytes at 22.900/mm^3^ (63% neutrophils). A chest radiograph showed an apical right lung consolidation. An abdominal ultrasound revealed an 8.4 x 9.0 cm right-solid renal mass. She was referred to the oncology clinic because a malignancy was suspected. No acid-fast bacilli (AFB) were seen on samples obtained from a gastric aspirate and a lumbar mass secretion. Erythrocyte sedimentation rate (ESR) was 105 mm/h, C-reactive protein was 25 mg/dL, and blood and urine cultures were negative. Family history was positive for TB in two siblings of 21 and 25 years of age, respectively, who had been on antituberculous treatment in the previous two months. There was no history of recent thoracic or abdominal trauma. 

Further laboratory investigations revealed normal blood urea nitrogen, creatinine, electrolytes, and urinalysis. The alanine transaminase (ALT) was 66 IU/L, serum aspartate aminotransferase (AST) was 222 IU/L, and albumin was 2.2 g/dL. A nasopharyngeal washing aspirate for respiratory viruses was negative by direct immunofluorescence test. An enzyme-linked immunosorbent assay (ELISA) test for human immunodeficiency virus (HIV) was negative. A repeat chest radiograph showed a right lung apical consolidation, a small pleural effusion, and moderate cardiomegaly. A repeat abdominal Doppler ultrasound revealed a dense and heterogenic avascular mass involving the right kidney entirely, with surrounding inflammatory reaction and invasion into the soft tissues. 

A lumbar mass puncture was performed, a small amount of fluid was obtained, and samples were sent for stains and cultures. Cefotaxime and clindamycin were started intravenously. Bronchoscopy revealed no structural or anatomical abnormalities. A bronchoalveolar lavage sample was sent for aerobic, anaerobic, fungal, and mycobacterial cultures. A purified protein derivative (PPD) skin test was negative. An echocardiogram showed a normal structural heart and moderate cardiomegaly, which was due likely to the anemia and hypoalbuminemia. A voiding cystourethrogram was normal. An abdominal enhanced computed tomography (CT) scan showed an infiltrative, dense mass that invaded the right kidney and extended into the soft tissues, including the fascia and muscles of the chest and lateral abdominal wall. Pleural effusion and thickening were documented by CT (Figure 1b). 

One week after admission to our hospital, she was taken to the operating room where severe destruction of the right renal parenchyma and extense renal adhesions to the surrounding structures, including the colon and the diaphragm, were found. Therefore, a complete right nephrectomy was performed. One day after surgery, an extense right pleural effusion was documented by a chest radiograph. A thoracocentesis was done which revealed exudate fluid, and a chest tube was inserted for one week. Two days after surgery, sulfur granules compatible with *Actinomyces sp.* were seen on histopathologic analysis of the renal specimen (Figure 1c), and no caseous necrosis or AFB were seen. The antibiotic therapy was changed to intravenous (IV) sodium penicillin (100,000 U/Kg/day, given q/6 hours). One week after, histological confirmation of actinomycosis was made from the surgical specimen. Tissue cultures were sterile for this and other microorganisms.

She made an excellent recovery and was transferred to a community hospital for completion of a four-week regimen of IV sodium penicillin, after which she was discharged home with oral penicillin for 12 months. A chest radiograph was normal two months later.

## Discussion

Multiple different clinical features of actinomycosis have been described, and various anatomical sites, such as the face, bone and joint, respiratory tract, genitourinary tract, digestive tract, central nervous system, skin, and soft tissue structures, can be affected. Actinomycosis frequently mimics malignancy, TB, or nocardiosis [1].

Cervicofacial actinomyces is the most common manifestation of an unusual infection. Its presentation is a suppurative infection with sinus tracts draining sulfur granules, mostly after dental manipulation. Pathogenesis involves mucosal injury and direct tissue invasion [2]. The organisms usually spread by direct extension but hematogenous and lymphatic dissemination may occur as well. 

The thoracic form often occurs secondary to aspiration. In this child, this was presumably the route of infection to the lungs, followed by hematogenous seeding to the kidney, and direct extension to adjacent soft tissues. Although we could not recover the organism from the lungs, we consider that this was the most likely etiology since the lung consolidation disappeared after two weeks of penicillin G treatment and no evidence of pleural effusion secondary to hypoalbuminemia or other infectious causes was found in her case. In this patient, it was important to include TB in the differential diagnosis because she lived in an endemic area and had also two siblings with the disease. The clinical and epidemiologic picture suggested TB, except for the fact that *Mycobacterium tuberculosis *does not invade across tissue planes as actinomyces and Nocardia do. The thoracic form is also associated with neurological or alcoholic disorders [4]. 

Abdominal actinomycosis is a rare infection, which is almost always misdiagnosed for disseminated malignancy or inﬂammatory bowel disease. Because of its low incidence and low suspicion rates, preoperative diagnosis is made in less than 10% of cases [5]. Abdominal actinomycosis usually occurs by the invasion of perforated, breached, or necrotic tissue [3]. 

Genitourinary actinomycosis is a very rare infection. Clinical ﬁndings are nonspeciﬁc and may include abdominal pain, urinary frequency, or repetitive cystitis. Generally, there is a long-term interval between the onset of symptoms and diagnosis. The diagnosis is suspected with histological findings since cultures are often unsuccessful, being positive in less than 50% of cases, as in our patient [6]. 

In our review of the literature, there were few reports of genitourinary actinomycosis:

Wacharachaisurapol et al. reported the case of an 11-yr-old girl who presented to the emergency department with six weeks of intermittent right hip and leg pain, three days of abdominal pain, and a swollen, indurated, tender, darkened area on her right lower abdomen that began draining bloody, yellowish, foul-smelling liquid [3]. She had also lost four pounds over a month's time. The patient was empirically treated with piperacillin-tazobactam and was taken into the operating room (OR) for incision and drainage. The wound cultures grew a few *Actinomyces meyeri*.

Niknejad et. al reported a case about an eight-year-old boy who presented with fever, weight loss, and flank pain two weeks after a sore throat [7]. The imaging studies were in favor of a malignant tumor of the right kidney, so he underwent a right radical nephrectomy. The pathologic study revealed xanthogranulomatous pyelonephritis and actinomyces bacterial colonies. Actinomycosis is a rare cause of xanthogranulomatous pyelonephritis in which individuals of any age from eight to 80 years could be involved. It is usually misdiagnosed as a malignant neoplasm in imaging studies [7]. It is important to know that renal *Actinomyces *is rare and it is difficult to recognize the infection. It should be considered in the etiological diagnosis for emphysematous and xanthogranulomatous pyelonephritis [8].

In a case study by Bianchini et al., a seven-year-old Italian boy was referred to a secondary-level hospital because of abdominal pain and dysuria [9]. Upon physical examination, a left hypochondrial mass was palpable. Ultrasonography showed a “hypoechogenic vascularized mass with undefined borders and a small amount of retrovesical fluid.” With magnetic resonance imaging, the doctors noticed that the mass extended from the left wall of the urinary bladder, which appeared enlarged and irregularly shaped. Considering a diagnosis of possible pelvic rhabdomyosarcoma, a transverse skin incision was made and multiple biopsies of the mass were performed. The biopsy cultures revealed *E. coli.* The patient received ceftriaxone for 14 days. The mass did not decrease in size; therefore, through a laparoscopic exploration, a frozen pelvis was documented and the dome of the bladder was partially excised. Histopathology of the excised bladder wall showed *Actinomyces*. 

Physicians should keep in mind not only the typical clinical presentations but also remember that actinomycosis may mimic a malignant disease in various anatomical sites. Bacterial cultures and pathology are the cornerstones of diagnosis, and typical microscopic findings, including necrosis with yellowish sulfur granules and filamentous Gram-positive fungal-like pathogens, are important to make the diagnosis [1].

Patients with actinomycosis require prolonged (six to 12 months) high doses of penicillin G or amoxicillin. However, the duration of antimicrobial therapy can be reduced to three months for patients in whom optimal surgical resection of infected tissues has been done [1]. Nephrectomy is not always necessary if the diagnosis of actinomycosis is made prior to surgery. In this particular case, the surgeons decided to perform a nephrectomy because of the lack of normal kidney parenchyma and profuse bleeding.

## Conclusions

Herein, we describe the case of a five-year-old female child with thoracic and renal actinomycosis who required both medical and surgical management. Pediatric actinomycotic is rare, accounting for less than 3% of all actinomycosis cases; 20% of pediatric cases occur in the abdomen. Actinomycosis frequently mimics malignancy, tuberculosis, or nocardiosis and can spread continuously and progressively. Renal actinomycosis, though uncommon, must also be considered in the differential diagnosis of renal masses and renal papillary necrosis. A high index of suspicion may help in prompt diagnosis and management, thereby preventing nephrectomy.
